# Simultaneous monitoring of potassium, glucose and lactate during spreading depolarization in the injured human brain – Proof of principle of a novel real-time neurochemical analysis system, continuous online microdialysis

**DOI:** 10.1177/0271678X16674486

**Published:** 2016-01-01

**Authors:** Michelle L Rogers, Chi Leng Leong, Sally AN Gowers, Isabelle C Samper, Sharon L Jewell, Asma Khan, Leanne McCarthy, Clemens Pahl, Christos M Tolias, Daniel C Walsh, Anthony J Strong, Martyn G Boutelle

**Affiliations:** 1 Department of Bioengineering, Imperial College, London, UK; 2 Department of Basic and Clinical Neuroscience, King's College, London, UK; 3 King's College Hospital NHS Foundation Trust, London, UK

**Keywords:** On-line microdialysis, microfluidics, neurometabolic coupling, spreading depolarization, ischaemic brain injury

## Abstract

Spreading depolarizations occur spontaneously and frequently in injured human brain. They propagate slowly through injured tissue often cycling around a local area of damage. Tissue recovery after an spreading depolarization requires greatly augmented energy utilisation to normalise ionic gradients from a virtually complete loss of membrane potential. In the injured brain, this is difficult because local blood flow is often low and unreactive. In this study, we use a new variant of microdialysis, continuous on-line microdialysis, to observe the effects of spreading depolarizations on brain metabolism. The neurochemical changes are dynamic and take place on the timescale of the passage of an spreading depolarization past the microdialysis probe. Dialysate potassium levels provide an ionic correlate of cellular depolarization and show a clear transient increase. Dialysate glucose levels reflect a balance between local tissue glucose supply and utilisation. These show a clear transient decrease of variable magnitude and duration. Dialysate lactate levels indicate non-oxidative metabolism of glucose and show a transient increase. Preliminary data suggest that the transient changes recover more slowly after the passage of a sequence of multiple spreading depolarizations giving rise to a decrease in basal dialysate glucose and an increase in basal dialysate potassium and lactate levels.

## Introduction

Traumatic brain injury (TBI) and ischaemic brain injury (occlusive and haemorrhagic stroke, aneurysmal subarachnoid haemorrhage, aSAH) are major public health problems. In the United States, it is estimated that 235,000 patients with TBI require admission to hospital each year, of whom some 50,000 die. Approximately 5.3 million Americans live with disability related to TBI.^1^ In high-income countries, TBI is the leading cause of mortality and disability in young people, creating a public-health problem with an estimated direct and indirect cost of $60 billion in 2000. A large problem with TBI treatment is the heterogeneity of the injury, with a range of pathologies of varying severity. The initial impact to the head causes a primary injury in the brain manifesting itself in, for example, intracerebral haemorrhage, subarachnoid haemorrhage, cerebral contusion, diffuse axonal injury, alone or in combination. For TBI, the core of, principally, a contusional injury can affect the nearby tissue, often reducing blood flow substantially, compromising neuronal activity and resulting in an array of cellular processes that are triggered by the nearby trauma, creating a peri-contusional zone at high risk of deterioration.^2,3^ Deterioration in approximately 40%^4^ of patients can lead to a secondary injury, whereby a larger area of brain tissue is affected. Causes of secondary injury after TBI can include ischaemia, cerebral hypoxia, cerebral oedema (either cytotoxic or vasogenic^5^) and raised intracranial pressure. Secondary deterioration is equally common after ischaemic brain injury of whatever oetiology, with similar causes, but possibly a greater relative role for spreading depolarizations (SDs; described below). Because of the problems of monitoring the metabolic state of the tissue in intensive care, there are limited targets available for potential treatments.^6^ However, the recent microdialysis consensus paper^7^ states that monitoring the brain chemistry, especially glucose, lactate and the lactate/pyruvate ratio, can give real insight into pathological mechanisms in the injured human brain.

At a tissue level, ischaemia is associated with alterations in the glucose and lactate levels as a result of disrupted aerobic metabolism. Astrocytes and neurons take in glucose from the extracellular fluid and microcirculation, and release lactate into the microenvironment during repolarisation of the membrane potential and, during prolonged periods of ischaemia astrocytes alone can produce lactate when delivery of glucose is insufficient.^8^ It has been hypothesised that lactate could serve as an alternative energy substrate for neurones.^9,10^ An elevated lactate/glucose ratio has been observed during ischaemic events^11,12^ and an elevated lactate/pyruvate ratio has also been associated with a poor patient outcome,^13,14^ as has low brain tissue glucose.^15^

SDs are a continuum of dynamic secondary insults to the injured brain that range from spreading depression (first recognised by Leao in 1944^16^) to anoxic depolarization (also described by Leao in 1947^17^). SDs are mass depolarizations of neurones and glia and are most simply detected in the human brain by electrocorticography (ECoG) using strip electrodes^18,19^ introduced at craniotomy, or using Spencer depth electrodes^20^ placed via a cranial access bolt. The self-propagating SD leads to a near-complete breakdown of ion homeostasis, causing a dramatic rise in extracellular potassium and a disruption in the cortical function and blood flow.^21–23^ A huge energy demand is placed upon the tissue to support repolarisation, to which the normal response is a brisk hyperemia. However, in injured brain, this response is often reversed to one of vasoconstriction (Figure 6 in Lauritzen and Strong, this issue), and the spread of depolarization is now closely accompanied by ‘cortical spreading ischaemia'.^21,24–27^ Spreading ischaemia is a complex phenomenon, and a sustained period can lead to tissue necrosis.^21^

SDs were first unequivocally observed as ECoG amplitude suppressions spreading in the human brain in 2002^18^ and are widely understood to represent an abnormal and spontaneous wave of mass depolarization of all cells, typically travelling through the tissue at 2–3 mm/min. SDs have since been shown to occur in 50–60% of TBI patients and have been noted to have yet higher incidences in other types of brain injury.^19,28–30^ When SDs occur adjacent to the injury core a repetitive clustering of events is often observed.^30^ A study by Murray et al. showed that the commonly logged clinical covariates such as age, pupil reactivity, level of hypoxia and motor score, explain only 30% of the variance in TBI outcome.^31^ Results obtained by the Co-Operative Study of Brain Injury Depolarizations (COSBID) group show that the occurrence of SDs independently predicts poor patient outcome and can enhance existing predictive models.^28^ As such, there is a need to further understand the biochemical processes occurring within the at-risk tissue that can affect patient prognosis. Multimodal monitoring can give an insight into such processes.

Classical microdialysis utilises individual samples and when used clinically, samples are typically collected hourly. This approach has not been capable of resolving the changes caused by a single SD, but glucose decreases and lactate increases have been recorded if a series of SDs occurred within one sample collection.^32^ A strong correlation between the number of SDs and the fall in local glucose levels has also been reported.^33^ On-line microdialysis has many advantages,^34^ including temporal resolution. Our previous approach to on-line microdialysis, rapid sampling microdialysis (rsMD), had 1-min resolution.^35^ This system could measure glucose decreases and lactate increases in experimental models of SD following stimulation in normal brain^36^ or occurring spontaneously in injured brain.^8,37^ With continual improvement, rsMD was able to detect the magnitude of the glucose falls and lactate increases occurring spontaneously in the injured human brain.^38^ However, the detailed time-course of changes could not be determined.^8,37,39^ The analysis system discussed in this article is also coupled to a microdialysis probe, but has been miniaturised using microfluidic techniques that have been designed for bedside analysis of key molecules indicating tissue health.^40^ An early version of the microfluidic system was used to measure SD in the rat brain^41^ using extracellular potassium concentration as a chemical marker for depolarization, and in experimental models, the potassium increase has been well established using micro ion-selective electrodes (ISE).^42^ We have monitored the metabolic effects of a variety of injury types affecting patients on the intensive care unit (ICU) and compared our findings from continuous online microdialysis (coMD) with those based on electrocorticography techniques, as well as with commonly recorded data such as blood pressure, intracranial pressure and PbO_2_.

## Clinical methods

### Patient recruitment and consent

Patients requiring emergency craniotomy for TBI, aneurysmal subarachnoid haemorrhage (aSAH), intracranial haematoma (ICH) or malignant hemispheric stroke (MHS) were identified and research consent obtained from relatives or the authorised surrogate. Patients under 16 years of age or with a Glasgow Coma Score below 4 at admission or with bilateral fixed and dilated pupils were excluded from this study. As patients were comatose at time of inclusion, written assent for study participation was obtained from legally authorised representatives. Written consent was obtained from the patients themselves once capacity had been re-established at follow up. All study data were anonymised and securely stored. All human research procedures were approved by the King’s College Hospital NHS Foundation Trust Ethical review board and were conducted in accordance with the Declaration of Helsinki.

### Electrocorticography

Electrocorticography (ECoG) data were collected as previously described.^20^ Briefly, at the conclusion of surgery a six-platinum-contact ECoG recording strip (Wyler TS06R-AP10X-0W6, 2.3 mm exposed diameter contacts with centres spaced at 10 mm along the strip, Ad-Tech Medical, Racine, WI, USA) was placed subdurally on the surface of the cortex to allow monitoring of viable tissue at risk of secondary injury.^19^ In TBI patients, strips were placed in cortical regions estimated to be peri-contusional penumbra at surgery. Silver/silver chloride (Ag/AgCl) scalp electroencephalographic (EEG) cup electrodes served as ground and reference. Data were acquired through Octal bioamplifiers (ADInstruments, Sydney, Australia) connected in a unipolar common reference configuration, with a lower frequency limit of 0.02 Hz and sampled at 1 kHz with 16-channel Powerlab analogue-to-digital converters (ADInstruments, Sydney, Australia) and recorded with LabChart software version 7.2 (ADInstruments, Sydney, Australia). Data from a micro movement sensor (MMS/TP, Unimed, Farnham, UK) were used to monitor patient movement and were also recorded and time-locked to the ECoG data using PowerLab and LabChart.

### Microdialysis probe location

Towards the completion of surgery, a sterile clinical microdialysis catheter (CMA 70, 10-cm flexible shaft, 10-mm membrane length, 20-kDa cutoff, CMA, Stockholm, Sweden) was inserted obliquely to the surface and to the full membrane length to ensure as far as possible that the catheter membrane was located within the cortex. The aim was to place the microdialysis probe close to the ECoG strip electrode and if possible on the same gyrus. Typically the microdialysis probe was located between contacts 4 and 5 of the ECoG strip and location was confirmed using a postoperative CT scan. Placement of the microdialysis catheter in the peri-contusional zone around the core injury is well accepted as the optimal location to detect early markers of deterioration in the ‘at-risk' tissue using this method.^43^ ECoG and microdialysis probes were secured by double suturing onto the skin beside the point of exteriorisation.

### Postoperative care in the ICU

Arterial blood pressure and intracranial pressure were continuously recorded and blood gases, glucose and electrolytes were documented periodically. All patients were treated with endotracheal intubation and were sedated principally with fentanyl and midazolam, which was replaced with propofol during withdrawal. Target cerebral perfusion pressure was 70 mmHg, applied flexibly.

## Analysis methods

### Online microfluidic analysis

**Figure 1.**
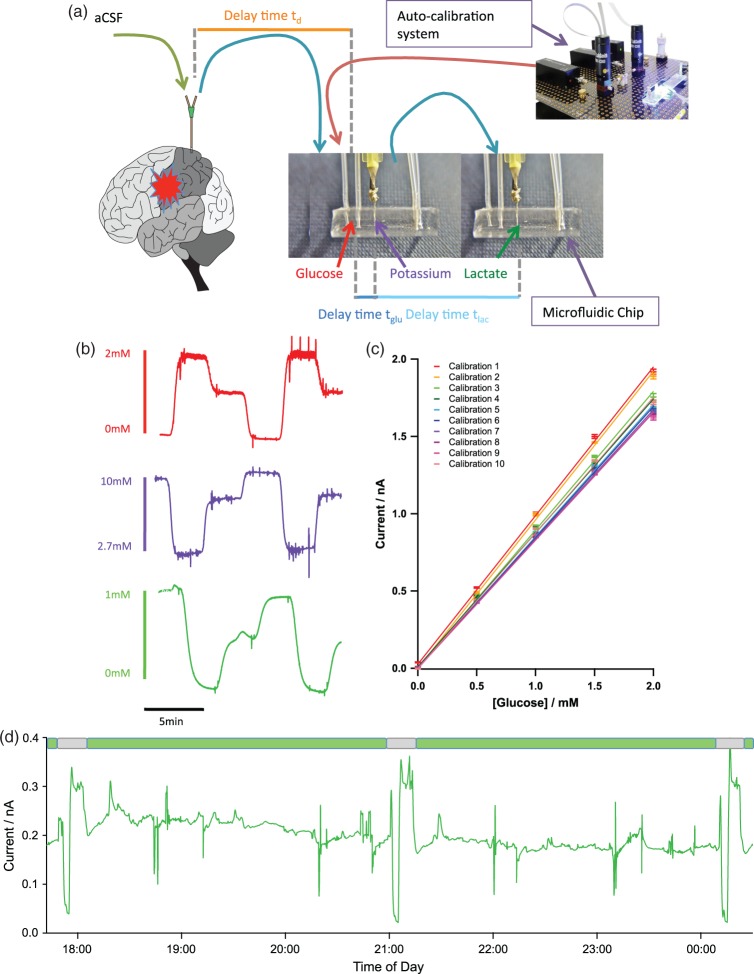
Continuous online microdialysis analysis system for bedside monitoring using microfluidic chips containing biosensors for glucose and lactate and a potassium ion selective electrode. (a) shows the overall setup. (b) Raw traces from glucose (red), potassium (purple) and lactate (green) during a computer-controlled three-point automatic calibration run. Concentrations indicated by legend. (c) Sequential analysis of sensor performance over 12 h using automatic calibration. (d) Raw data for microdialysate brain lactate levels collected at the bedside with three automatic calibrations. The green boxes indicate sections of clinical data and the grey boxes indicate calibrations. Clinical data were collected from patient 2.

The microdialysis catheter was perfused with sterile artificial cerebrospinal fluid (aCSF) (CMA perfusion fluid: 147 mM NaCl, 2.7 mM KCl, 1.2 mM CaCl_2_, 0.85 mM MgCl_2_) at 2µL/min using a CMA 107 microinjection syringe pump (CMA Microdialysis, Stockholm, Sweden). Perfusion of the microdialysis catheter was started immediately, so that the initial baseline dialysate levels recorded in the ICU had stabilised substantially. The outlet tubing of the probe was adapted to connect to our online microfluidic analysis system. Typically a 1 m length of low-volume connection tubing (Microbiotech, Stockholm, Sweden) was used between the microdialysis catheter outlet and the online assay to facilitate patient movement and nurse care. The microdialysate was perfused through a microfluidic chip containing sensors to detect glucose, lactate and potassium levels. The setup can be seen in Figure 1.

Glucose and lactate sensors were fabricated onto a needle microelectrode design described elsewhere.^44^ Briefly, Teflon insulated 50 µm platinum wire (A-M Systems Inc.) and polyester insulated 50 µm silver wire (AM systems) were threaded through a hypodermic needle. The platinum disc acted as the working electrode and the silver disc was chloridised using a referencing solution (BASi Inc) to create a Ag/AgCl reference electrode. The needle shaft acted as a counter electrode. The needle electrode was polished using alumina slurries and cyclic voltammetry was used to assess the surface of the working electrode. All the chemicals are obtained from Sigma-Aldrich (UK) unless otherwise stated. To fabricate the biosensors for glucose and lactate, a protective m-phenylenediamine (mPD) film (100 mM in phosphate buffered saline, pH = 7.4) was electropolymerised onto its surface. The presence of a mPD film sufficient to protect against interference by electroactive species in the dialysate was tested for each biosensor using cyclic voltammetry. A second layer adds specificity for glucose and lactate, and consists of a hydrogel loaded with enzyme that is dip coated onto the needle electrodes.^45^ This polyethylene glycerol hydrogel matrix contained entrapped substrate oxidase (either glucose oxidase or lactate oxidase).

Potassium sensors were fabricated based on a miniaturised ISE design. An ISE features an ion-sensing membrane that selectively binds to the ion of interest and its output potential is proportional to the activity of the ion. A polymer membrane cast at one end of a polymer electrode body (perfluoroalkoxyalkane tubing), an internal Ag/AgCl reference electrode and an internal filling solution of physiological saline. The membrane consisted of potassium tetrakis(4-chloropheyl)borate, bis(2-ethylhexyl) sebacate, poly(vinyl chloride) and the potassium ionophore, valinomycin. The assembled ISEs were stored in aCSF at 4℃ when not in use.

The three sensors were housed on a microfluidic platform designed for online microdialysis monitoring. Briefly, the microfluidic chips were fabricated using soft lithography techniques. First, a negative master made from SU8 (Microchem) was generated using 1:1 contact photolithography. The PDMS chip is made by thoroughly mixing PDMS base and curing agent (Sylgard 184 elastomer 10:1 ratio by weight) and baking for 1 hour at 65℃. Once cured, access holes were punched and the chips were placed channel side down onto a semi-cured PDMS base. The top and bottom layers were sealed together by baking at 65℃ overnight. The complete system is shown schematically in Figure 1(a).

The oxidase-based biosensors used in this system rely upon molecular oxygen as a mediator. Although oxygen changes are seen in the human brain, especially during SDs, any effect on the sensor and the recordings was eliminated due to the high permeability of PDMS and FEP tubing to oxygen. As both glucose and lactate biosensors depend upon the oxidation of hydrogen peroxide produced during the enzymatic reactions at the electrode surface, there was a possibility that cross talk could affect the signals recorded at the downstream sensor. However, the system was protected against this effect in several ways. First, the order of the sensors within the flow system was such that the predicted concentration changes reduced the effect of cross talk, i.e. glucose was upstream of lactate because the dialysate level of glucose was low and, during SDs, it is likely to be pushed to lower concentrations, whereas the lactate level in the dialysate is typically higher and increases in concentration were predicted to be observed. Second, at the flow rate used here, 2 µL/min, the amount of hydrogen peroxide that is produced by the microelectrodes and carried downstream is too small to be detected. This was tested in the laboratory prior to use on the clinical ward.

### Automatic sensor calibration

We have developed a novel auto-calibration system using a series of programmable valves and microfluidic pumps (LabSmith, California, US). These are arranged in such a way as to deliver multiple standard concentrations to calibrate the on-line sensors without disrupting the perfusion and recovery of the microdialysis catheter. In this setup, the dialysate flow could be diverted into a collection vial so that a constant flow and recovery across the probe membrane was maintained. Critically, in this manner, no artefacts in the data are produced. A series of various known calibration solutions could then be passed into the microfluidic analysis system, at chosen times (typically every 3 h). Glucose standards are perfused from a high to low concentration and lactate and potassium are simultaneously perfused at a low to high concentration. During setup, there are times when only one sensor is running. Here, we can ensure that there is no crosstalk effect when all sensors are run together. Figure 1(b) shows a typical calibration of glucose (red), potassium (purple) and lactate (green). The auto-calibration system allows the sensitivity of the sensors to be tracked over time, as seen in Figure 1(a) and (d), ensuring that each block is surrounded by recent calibrations facilitating reliable interpretation when the data are reported and analysed. Typically, the sensors are changed daily ensuring that a functional screening layer is always present and that the sensors have sufficient sensitivity and time responses.

### Complete clinical system

The microfluidic analysis system was placed on a clinically certified trolley (Bristol Maid, Blandford Forum, Dorset), which was located behind the patient bed in the ICU to cause minimal disruption to clinical care for the duration of the study. The sensors were controlled via a lab-built potentiostat^46^ feeding into the Powerlab running LabChart Pro (AD Instruments). The data were recorded continuously for a minimum of 24 h and a maximum of 5 days, Table 1.

**Table 1.** Demographics of patients monitored using coMD.

Patient	Injury	K + events	Glu events	Lac events	SD events	MD working? Comments
1	TBI, SDH, SAH	4	4	4	1	✓
2	ICH	8	8	8	0	✓ Probe located in a different area
3	MHS	8	8	8	8	✓
4	SAH	8	8	8	4	✓No ECoG available
5	TBI, SDH	6	6	25	4	✓Timing issues
6	TBI, SDH	1	1	0	1	✓No ECoG at the event time point

**Figure 2.**
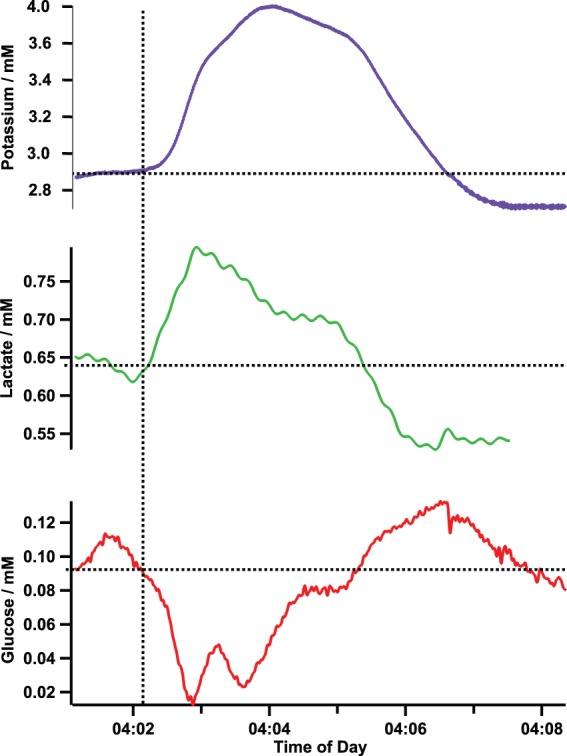
Spreading depolarization recorded in patient 5 confirmed by ECoG. The increase in potassium indicates the depolarization of the cells surrounding the microdialysis probe. The fall in the local concentrations of glucose and the rise in the level of lactate indicates that the demand for energy is outstripping the supply, thus creating a mismatch that is potentially harmful to the local tissue area.

The number of physiological events is noted in each data channel before being collated and assessed for evidence of SD events. Whilst the microdialysis dataset of patient 2 contained eight physiological transients, they do not follow the expected trend for an SD wave, shown in Figure 2. We suspect that these are responses to other physiological events, which we are investigating further and that are outside the scope of this paper.

### Data analysis

The results presented from the sensors have been time-aligned to each other (based on the time delays, *t_glu_* and *t_lac_* in Figure 1(a), observed during calibration). The microdialysis study data were then time-aligned to the ECoG data by accounting for the transit time down the 1-m length low-volume connection tubing between the patient and the analysis (time delay *t_d_* in Figure 1(a)). The microdialysis data were then screened for changes of physiological origin such as arterial blood pressure, or intracranial pressure. In separate in vitro experiments, we determined the 90% response time of the analysis system (including microdialysis probe and 1-m connection tubing) to be 68 s. This compares to 5 s for the sensors in free solution.

### Improvements to data quality

Initially, noise from the busy intensive care environment lowered the sensitivity of the assay, affecting the data in the earlier patients. There was a marked difference between the lab environment and the clinical environment in terms of electrical interference. Ongoing work has screened out as much of the electrical interference as possible, with good improvements.

Another factor affecting the earlier microdialysis monitoring was the placement of the probe in relation to the other probes implanted. In some cases, the probe was positioned in a separate area of the brain, sometimes on a different gyrus. The interpretation of the data collected from these patients was very difficult as they could not easily be time-aligned. In some cases, such as patient 2, the microdialysis probe was located 8 cm away from the nearest contact on the strip electrode. In this case, there was a sulcus between the two recording methods so whilst SDs were noted at the strip electrode, the SDs did not necessarily travel towards the MD probe and we cannot know if they in fact crossed the sulcus. Time-aligning these two datasets was difficult and not without error. Assuming an SD travels through the cortex at 2–3 mm/min, the time interval between the two probes is larger than the interval between repeating SDs creating further complications. The protocol has since been changed to ensure that the microdialysis probe is located as near as possible to the ECoG strip, often with the microdialysis membrane located underneath the strip electrode between two of the contact points. In this way, comparison between the microdialysate data and the electrophysiology collected is more reliable.

## Results

Microdialysis data were continuously recorded together with EGoG data for periods of between 24 h and 5 days in six patients (Table 1). The passage of an SD (confirmed using ECoG) yielded a characteristic response signature in the microdialysis neurochemical levels, Figure 2.

The SD is reflected in the tissue surrounding the microdialysis probe by a transient increase in the potassium level, indicating depolarization. On the same timescale as the potassium changes (5 min in this case), there is a transient increase in lactate and decrease in glucose. This signature was seen at every confirmed SD that we have monitored using coMD in patients and in an experimental model in which SDs were induced.^41^ The huge energy demand, created by the need to repolarise cellular membranes, causes a decrease in the local glucose level due to the mismatch between energy supply and demand. The level of lactate in the tissue transiently increases, possibly reflecting anaerobic glycolytic metabolism of glucose. In this patient (patient 5), the basal level of glucose in the dialysate is low, at approximately 100 µM, despite blood glucose levels being high (11.8 mM). A lower level of brain glucose is detrimental due to the tissue being deprived of a key energy source, which if prolonged can lead to local tissue death. The SD reduces the local level of glucose in the tissue down to approximately 20 μM, a level that is critically low for tissue survival.

**Figure 3.**
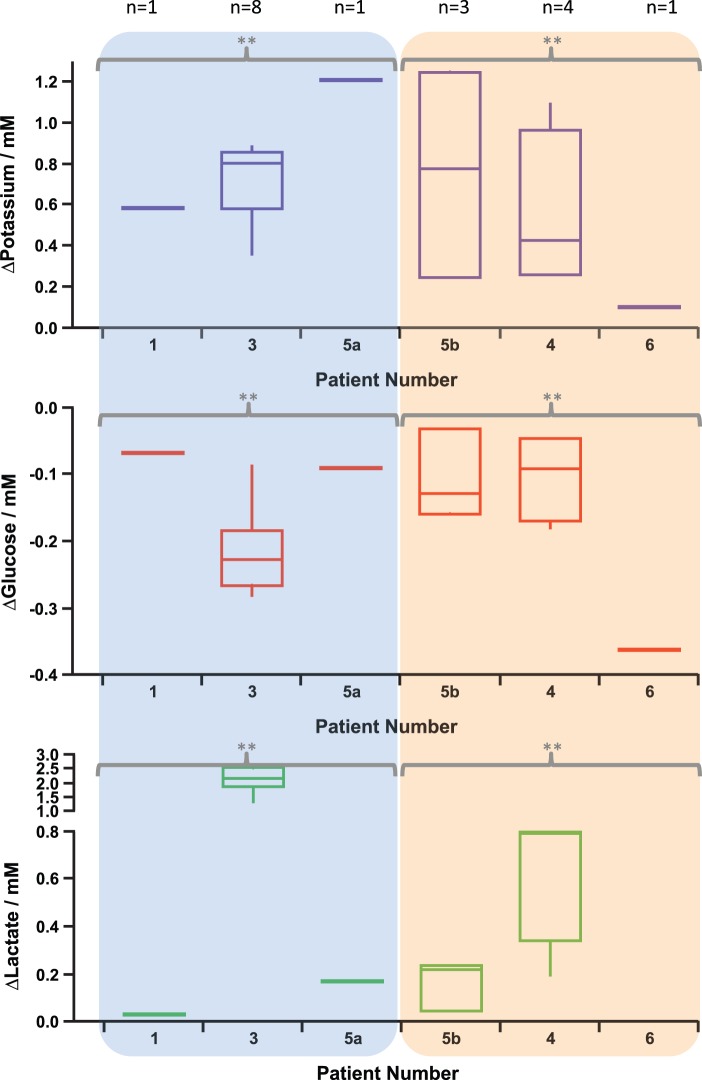
Microdialysate changes during spreading depolarizations. SDs that are confirmed using ECoG are seen in patients 1, 3 and 5a, as indicated by the blue-shaded box. In patients 4, 5b and 6, the events have not been confirmed as SDs, either as there was no ECoG data recorded at that time or due to problems time-aligning the two datasets. This is indicated by the orange-shaded box. In patient 6, there is no lactate data available at the time of the event. Significance of the change from baseline to peak was tested using a Wilcoxon signed-rank test, p = 0.01.

A combined total of 10 events in three patients displayed the SD microdialysate signature and were confirmed by electrophysiology, as shown in the first three columns of Figure 3. A further eight events showed this signature without ECoG confirmation, due to no ECoG monitoring or due to technical issues affecting timing, in three patient datasets. In patient 5, the three unconfirmed events were stereotypical when compared with the confirmed event within the same patient dataset. Unfortunately, in patient 6, the lactate sensor was not functioning during the period where the event occurred and the data were lost. Therefore, in Figure 3, there is no data within the corresponding lactate column.

The potassium transients showed variability in height and, particularly, in duration, which appears to impact the duration of the glucose and lactate transients. For this reason, the peak height has been analysed for each event rather than using integrative measures such as area-under-the-curve. Figure 3 shows the median change in a box and whisker plot. A Wilcoxon signed-rank test was conducted comparing the baseline before the event to the peak concentration and significance was found in all analytes at the 0.01 significance level in both confirmed and unconfirmed events.

**Figure 4.**
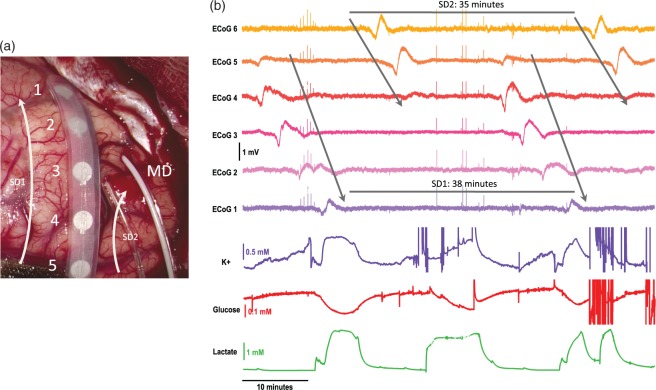
ECoG and microdialysate data of repetitive spreading depolarizations collected from patient 3. (a) Image of probe placement on the brain taken during craniotomy. The numbers relate to the ECoG channels and the arrows to the potential paths of two SD waves. (b) Data showing a total of four SD waves. Top six traces show the large slow potential change in the near-direct current (DC) ECoG data, a hallmark characteristic of SD. The bottom three traces show the tissue response from the microdialysate data: potassium (purple), glucose (red) and lactate (green). The arrows indicate the SD waves in the ECoG data. There are two waves repeating with cycles of 38 and 35 min.

Data from patient 3, who suffered a malignant hemispheric stroke (a patient group with a high likelihood of SD,^25,29^ are shown in Figure 4. In Figure 4(a), the probe positions can be seen, with the microdialysis probe located in the cortex at an acute angle. Figure 4(b) shows the data from the electrical contacts of the ECoG strip and the data collected from the microdialysate; potassium, glucose and lactate levels. There are multiple SD waves occurring in this segment of data, seen clearly in the ECoG data as indicated by the arrows. The metabolite response to the SD waves is shown below (microdialysate data).

There are in fact two SD waves repeating throughout this area of tissue. The waves have a similar periodicity, which is stereotypical of SD waves previously recorded.^19,30^ The predicted paths of the two waves are indicated by the arrows in Figure 4(a). One wave travels down the full length of the ECoG strip electrode and the second wave travels obliquely across the ECoG probe. Both waves are recorded in the MD data.

With each SD, the potassium levels transiently increase as all the cells surrounding the microdialysis probe depolarise. The glucose levels transiently fall, and lactate levels transiently rise, indicating that the cells are becoming ischaemic suggesting a shift to anaerobic glycolysis during repolarisation. Unfortunately, the calibration (set to run automatically every 3 h) occurred in the microdialysis system at this time, causing the fourth SD seen in the ECoG data to be lost from the microdialysis data. The glucose and lactate changes seen here are large compared with other patients in this study and to those measured previously using rsMD.^38^ The patient’s blood glucose level at this time was high, at 14 mM. It has previously been noted that at higher blood glucose levels, the glucose and lactate responses tend to be larger.^38^

**Figure 5.**
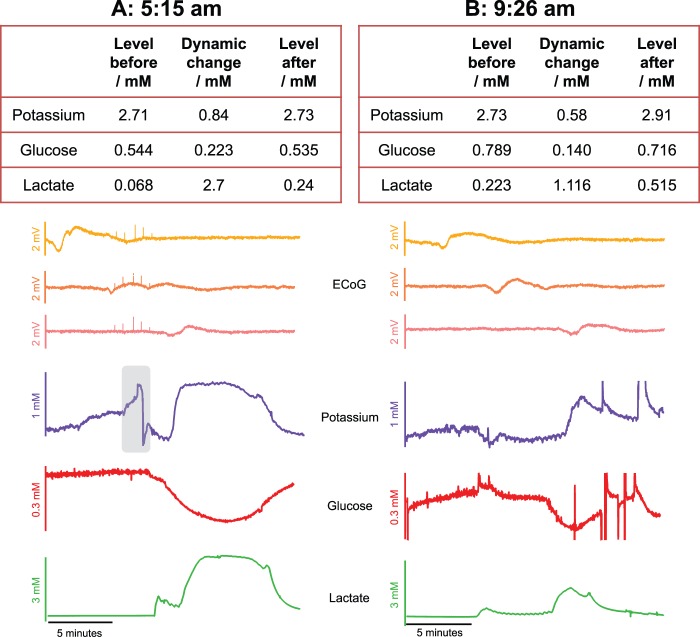
Spreading depolarizations occurring over 4 h apart within the same patient dataset (patient 3). (a) Data of an SD occurring at 5:15 am. The table shows levels of potassium, glucose and lactate before and after the event and the traces show the data. Note that only three traces of the near-DC ECoG are shown for simplicity. The grey box represents an electrical artefact in the baseline potassium level that was not included in the baseline measurement. (b) Data of an SD occurring over 4 h later at 9:26 am. The table shows levels of potassium, glucose and lactate before and 10 min after the event and the traces show the data. Note that only three channels of the near-DC ECoG data are shown for simplicity.

The first event seen in Figure 4(b) is shown in more detail in Figure 5(a). For simplicity, only three of the ECoG data channels are shown (channels 1, 2 and 3) for each event. The baseline levels for potassium, glucose and lactate (2.72, 0.544 and 0.068 mM, respectively) before the SD wave are typical microdialysate values monitored in brain injury patients. The dynamic changes observed in response to the SD wave are large and the levels do not return to the pre-event levels within 10 min. From this, we can infer that the tissue is not fully recovering from the SD insult. Figure 5(b) shows an SD event occurring over 4 h later in the same patient dataset. Here, the size of the change is less; however, the concentration changes are still substantial. During the SD, the level of potassium is pushed higher still and remains at this higher concentration; 10 min after the SD event occurs, the potassium level is 2.91 mM. Although the baseline level of glucose increased after the first event, the local concentration of glucose is still lowered during the SD. The lactate level follows the same trend and although the dynamic change is smaller than the first event, it is still a substantial concentration change. After the SD wave, the level of lactate remains high at 0.515 mM. Looking at the band-pass filtered (0.5 to 45 Hz) ECoG data, no depression was seen during these events because ECoG amplitude was already depressed, indicating that these events were in fact isoelectric SDs. The microdialysate signature indicates that the tissue has a higher energy demand during an SD, which is not being met by local blood supply.

## Discussion

The development of a coMD has allowed the recording of a neurochemical signature for SDs. The potassium profile during the course of an SD wave was first recorded in the rat brain using implanted microelectrodes^47^ and there is one previous report of recording potassium changes with a surface ISE during SDs in one patient.^48^ In these reports, resting extracellular potassium was 3 mM (rat), and most probably similar in the patient^48^: the (SD) peak potassium value was around 60 mM (experimental) and in the region of 30–60 mM (patient), but it is to be expected, given the microdialysis probe collection efficiency (approximately 20%) and the attenuating effect of Taylor dispersion down a 1-m length tube on a rapidly changing response,^34^ that the microdialysis potassium change seen with coMD will be substantially smaller than that detected by implanted microelectrodes. Even mildly elevated (30-min pooled samples) levels of dialysate potassium have been associated with a poor patient outcome^49^ and the ability to see real-time online changes in potassium concentrations not only allows a chemical marker of SDs to be recorded but also baseline (inter-SD) potassium levels may serve as a valuable neurochemical marker related to patient outcome. The transient changes in glucose and lactate furnish measures of the tissue’s ability to cope with the huge energy demand of repolarisation. The decrease in glucose associated with the SD wave indicates a mismatch between energy supply and energy demand of the tissue surrounding the probe. The transient increase in lactate possibly reflects anaerobic glycolytic metabolism of glucose. Measurement of these key metabolites has recently been presented as an important combination of neurochemical markers that provides information on local tissue viability.^7^ A criticism of microdialysis is the use of a single MD probe to report the neurochemical state of a wider, less well defined area of injured brain. This is mitigated for focal injuries by placing the probe in ‘at-risk' tissue adjacent to the core area of contusional damage or infarction.^7^ coMD confers the advantage of excellent temporal resolution, which is demonstrated when monitoring the tissue response to repetitive SD waves. The ability to document the passage of the SD, and the metabolic response of an SD, as the wave travels past the MD probe allows a characteristic signature to be established that describes the state of the tissue and its response to perturbations that may place it at risk. This is highly advantageous during a cluster of repeating SD waves, where the high time resolution allows each passing wave to be accurately measured, as opposed to a global measurement that loses information in the averaging of data. Clusters of apparently repetitive SDs can be attributed to recurrent cycling of a single SD wave around a core of permanently depolarised tissue,^50^ and on this basis, the chemistry of the sampled tissue can be cautiously extrapolated to the peri-contusion tissue more broadly. Repeated SDs have been shown to be detrimental to local tissue health, most probably by enlarging infarct/contusion size,^51–53^ and this may be indicated in potassium and lactate remaining above, and glucose remaining lower than, pre-event baseline levels.

The new findings confirm, at a higher temporal resolution, the lowering of brain glucose caused by SD that we had previously observed with rsMD in patients.^38^ A fall in tissue glucose levels to near zero (such as is shown in Figure 2) for prolonged periods of time has been associated with poor patient outcome.^15^ We have also previously shown in experimental models that SDs occur more frequently in the injured brain when glucose availability is low.^35^ This combination of findings raises again the possibility of a vicious circle in which SDs reduce brain glucose through intense metabolic demand, in turn increasing the likelihood of further SDs.

Work is ongoing to investigate the factors leading to variation in the magnitude and duration of the responses measured, and to present the signature together with events detected in ECoG and other parameters measured in our multimodal monitoring system as a real-time clinical visualisation tool.

## Conclusion

This article presents a novel monitoring system, coMD, which we have used to measure neurochemical changes in real-time in the injured human brain. It has allowed us to confirm in the human brain the dynamic signature of ionic and metabolic neurochemical changes caused by a SD at high time resolution. This is an important advance as it can allow the clinical care team to see not only that an SD has occurred but also what effect it has on the underlying neurochemical state of the brain tissue. We show that an SD is accompanied by a transient increase in dialysate potassium that is coupled, on the same timescale of a few minutes, with a transient fall in glucose and a rise in lactate levels. Together, these form a recognisable signature of ionic and metabolic changes associated with a SD.
